# Modelling HIV disease process and progression in seroconversion among South Africa women: using transition-specific parametric multi-state model

**DOI:** 10.1186/s12976-020-00128-5

**Published:** 2020-06-23

**Authors:** Zelalem G. Dessie, Temesgen Zewotir, Henry Mwambi, Delia North

**Affiliations:** 1 School of Mathematics, Statistics and Computer Science, University of KwaZulu-Natal, Durban, South Africa; 2 College of Science, Bahir Dar University, Bahir Dar, Ethiopia

**Keywords:** AFT models, Markov model, Latent variables, Factor analysis, Waiting probabilities, Transitions and quality of life domain scores

## Abstract

**Background:**

HIV infected patients may experience many intermediate events including between-event transition throughout their follow up. Through modelling these transitions, we can gain a deeper understanding of HIV disease process and progression and of factors that influence the disease process and progression pathway. In this work, we present transition-specific parametric multi-state models to describe HIV disease process and progression.

**Methods:**

The data is from an ongoing prospective cohort study conducted amongst adult women who were HIV-infected in KwaZulu-Natal, South Africa. Participants were enrolled during the acute HIV infection phase and then followed up during chronic infection, up to ART initiation.

**Results:**

Transition specific distributions for multi-state models, including a variety of accelerated failure time (AFT) models and proportional hazards (PH) models, were presented and compared in this study. The analysis revealed that women enrolling with a CD4 count less than 350 cells/mm^3^ (severe and advanced disease stages) had a far lower chance of immune recovery, and a considerably higher chance of immune deterioration, compared to women enrolling with a CD4 count of 350 cells/mm^3^ or more (normal and mild disease stages). Our analyses also showed that older age, higher educational levels, higher scores for red blood cell counts, higher mononuclear scores, higher granulocytes scores, and higher physical health scores, all had a significant effect on a shortened time to immunological recovery, while women with many sex partners, higher viral load and larger family size had a significant effect on accelerating time to immune deterioration.

**Conclusion:**

Multi-state modelling of transition-specific distributions offers a flexible tool for the study of demographic and clinical characteristics’ effects on the entire disease progression pathway. It is hoped that the article will help applied researchers to familiarize themselves with the models, including interpretation of results.

## Background

HIV is one of the leading causes of mortality amongst infectious diseases globally and is recognized as a main public health problem [1]. AIDS, the last disease progress stage of HIV infection, leads to severe damages to the body’s resistance system [1, 2]. Progression of HIV/AIDS is highly variable between individuals and populations and is determined by immunologic, environmental, genetic, and virology factors [3]. Improved awareness of the HIV disease process and progression pathway, including influencing can add great value to understanding HIV pathogenesis and developing treatment strategies [4].

CD4 cell count is the surrogate marker of HIV disease progression regularly used in the clinical setting to monitor the infection [5]. It is also an accurate marker of the stage of HIV disease and is recommended by all guidelines of HIV management [6]. Researchers also argue that the HIV viral load appears to be the best predictor of long term clinical outcomes, whereas CD4 cell count predicts clinical information (event time data) [7]. CD4 cell count has informed us when to start and stop opportunistic infection management or prophylaxis when to start antiretroviral therapy, OI risk stratification, as well as in monitoring response to treatment [8, 9]. Thus, CD4 cell count has been an important factor in the clinical investigation of HIV as well as used as a prognostic marker for assessing HIV disease process and progression.

HIV patients go through severe, advanced, mild, and normal clinical stages and *CD4 cell count* provides a biomarker for characterizing these *clinical stages* [6]. Also, the disease diagnosis can be considered as one of these clinical stages. Modelling of these sequential clinical stages may be a better capture of the complete HIV/AIDS disease progression. It is also essential to understand and predict accurately the course of the disease evolution, and it is of particular relevance for the clinicians that need to distinguish the different types of events in order to properly adapt the treatment.

For modelling the disease progression of HIV/AIDS, there are some specific challenges: there may be several intermediate events for the patients, from HIV infection to death; and some clinical variables may be measured over time and are therefore often not available for some cohort members. These challenges require a specific arrangement and preparation of the data for analysis as well as the specification of appropriate models, in order to allow for the cyclic patient longitudinal data. To address the above-mentioned challenges, we have presented multi-state transition-specific parametric models. This is an important point that has not been considered in HIV/AIDS cohort studies particularly in Sub-Saharan Africa. A multi-state transition-specific parametric model allows rich approaching into complex disease processes and progression pathways, where the patients may experience some intermediate endpoints, and in addition, the model permits the analysis to examine the possible covariate effects on each specific transition [10–12]. Multi-state models are very useful for describing event-history data, giving a deeper understanding of disease process and progression and how other patient’s demographic and clinical characteristics affect the entire disease progression pathway.

Most methodological developments have usually focused on semi-parametric methods, by using the Cox regression method as the basic framework of multi-state modelling [11, 13–16]. The fully parametric multi-state approach is less well known and somewhat of new development in medical data, where the same distribution is assumed for all transitions [2, 17]. Through a fully parametric survival model approaching, there are many advantages: predicting, modelling time-varying variable factors, gaining a better understanding of how factors change with time, etc. as compared to a semi-parametric method [18]. The basis of this paper is to present a multi-state model considering the requirement of distribution for each transition, approaching a variety of fully parametric models, and allowing for structures to be shared across the particular transitions. In some cases, there will be limited data for particular transitions and we may have an inclination to assume simple parametric distributions, for example, the exponential distribution; and while we may have more information for a specific transition, we may use a more complex parametric distribution [19]. Furthermore, to our knowledge, little research has emerged that examines the consequences of fitting transition specific distributions to multi-state models for medical data with time to sequential adverse events.

The purposes of this study are thus three-fold. Firstly, we seek to introduce the multi-state Markov modelling framework, briefly describing previous approaches. Secondly, we use a non-parametric method (Aalen-Johansen estimator) to estimate transition probabilities and length of stay in a particular state. Finally, we seek to develop multi-state models, which allow for transition-specific distributions and to apply this model to describe the complete evolution process of HIV in ART-naive individuals so as to aid in obtaining a deeper understanding of HIV disease progression and to discover possible factors that influence immune deterioration.

## Methods

### Study population

The data is from an ongoing prospective cohort study from the CAPRISA. CAPRISA initially enrolled HIV-negative (phase I) women into different study cohorts. The main study was a prospective cohort study (the CAPRISA 002), aimed at documenting acute infection with an extensive follow up to determine the natural history of the HIV-1 subtype C infection. The establishment of the CAPRISA 002 acute infection study was between August 2004 and May 2005 [20]. It was conducted at the Doris Duke Medical Institute (DDMRI) situated at the Nelson R. Mandela School of Medicine of the University of KwaZulu Natal in Durban, South Africa. Participants were recruited at two sites in KwaZulu-Natal: an urban site in Durban and a rural site in Vulindlela. Participants without well documented estimated date of HIV infection and those who were lost to follow-up during the observation period, were excluded in this analysis. Further information about these ongoing prospective HIV cohort studies, including women’s eligibility criteria, were reported in [20–22]. Finally, two hundred and nineteen (219) participants were included in the study.

### Variables and measurements

Once HIV diagnosis was confirmed, participants were enrolled into the acute HIV infection cohort and were then followed up for a maximum of 13 years at the time of this analysis. The follow-up assessment phase was classified as follows: acute infection (i.e. phase II: this was taken as follow up time within 0–3 months after infection), early infection (i.e. phase III: the time period during which the patients were followed up between 3 to-12 months’ post-infection), established infection (i.e. phase IV: It started from 12 months’ post-infection and ended once the patient has initiated antiretroviral therapy) and on cART (phase V: the patient was on ART- in this study, it was initiated when the CD4 cell count was below 500 cells/mm^3^). Samples for immunologic, virologic, and clinical parameters (such as viral load, CD4 counts, etc.) were measured at each visit [23]. There was a total of 8760 followed-up visits recorded from 219 HIV infected women.

The main outcome variable in this current paper is time to sequential adverse events. World Health Organization immunological classifications were used to assess the degree of severity of HIV infection of patients in the study. These HIV infection stages are defined as no adverse events (CD4 ≥ 500), mild (350 ≤ CD4 ≤ 499), advanced (200 ≤ CD4 ≤ 349) and severe (CD4 < 200) [6].

**Fig. 1:**
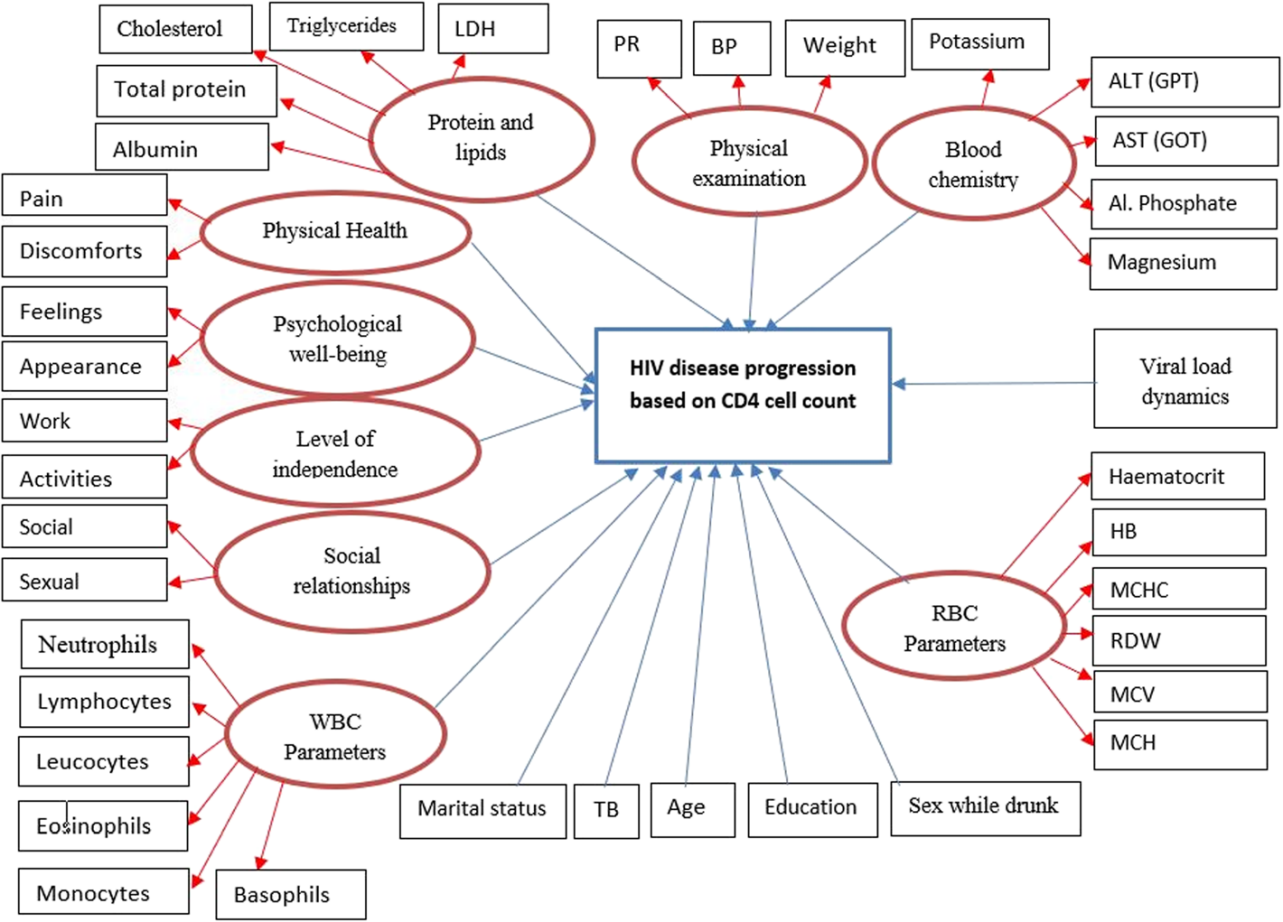
Graphical display of hypothesized model

The effect of several factors on time to sequential adverse events was evaluated including: (1) demographics: age, marital status, educational level, and sex under the influence of alcohol; (2) OI: hypertension and tuberculosis; (3) risk varaibles: substance use, contraceptive use, and sex under the influence of alcohol; (4) clinical parameters: WBC components (neutrophils, lymphocyte count, monocytes, eosinophils count, and leucocyte count), Blood chemistry (sodium, chloride, calcium, ALT, AST, total protein and LDH), RBC parameters (Hb, RDW, MCH, MCV, MCHC, and hematocrit) and (5) QoL domain scores. The QoL questionnaire [24], was used to measure QoL of HIV infected patients. Therefore, the QoL scales contain the following domain. The first is physical health scores, that assess perceived working capacity, lack of energy and initiative, fatigue, the presence of pain, dependence on therapeutic substances, and the impact of the disease on the activities of daily living. The second is the psychological-wellbeing score domain, which measures the patient’s thoughts about body appearance, negative and positive suicide, anxiety, higher cognitive functions, self-esteem and personal beliefs, feelings, depression, and spirituality. The third domain is social relationships, which measures sexual activity, social support, social contacts, and personal relationships. The fourth domain is devoted to the level of independence and measures areas such as work capacity, dependence on treatments, activities of daily living, and mobility. Further information about the above-mentioned factors was reported in [25, 26]. (See Fig. 1).

### Statistical method

#### Factor analysis

Since the dataset has numerous clinical parameters, we used the factor analysis (FA) methods to minimize and group the parameters. Exploratory FA was done by creating the principal components of the original variables and then creating the eigenvectors. By using the Kaiser-criterions, eigenvectors with eigenvalues greater than 1 were kept [27]. A maximum likelihood extraction method with varimax rotation was used. Each observation was assigned a score for each rotated factor, based on the loading of the subject’s original variable levels. Accordingly, we managed to group the 24 clinical variables in the study, to create 9 latent variables, defined as protein component, lipid component, electrolyte component, liver abnormality component, red blood cell indices, Hb and haematocrit component, eosinophils component, mononuclear component, and granulocytes component. (See Table 1).

**Table 1:** Clinical parameters and corresponding factor loadings from the rotated factors

Clinical parameters	Principal Components	Variables	Factor loadings	Commutative variations
White blood cell parameters	1. Granulocytes component	Leucocyte	**0.925**	77%
		Neutrophils	**0.936**	
		Monocytes	**0.635**	
	2. Mononuclear component	Lymphocytes	**0.838**	
		Basophils	**0.616**	
	3. Eosinophils component	Eosinophils	**0.947**	
Red blood cell parameters	4. Hb and haematocrit component	RBC counts	**0.946**	81%
		Hb	**0.886**	
		Haematocrit	**0.919**	
	5. RBC indices component	MCV	**0.953**	
		MCH	**0.825**	
		MCHC	**0.521**	
		RDW	**−0.592**	
Blood chemistry	6. Liver enzyme abnormality component	ALT (GPT)	**0.829**	72%
		AST (GOT)	**0.967**	
	7. Electrolyte component	Chloride	**0.455**	
		Sodium	**0.994**	
		Calcium	**0.213**	
Protein and lipids	8. Lipid component	Cholesterol	**0.971**	65%
		LDL	**0.917**	
		Triglycerides	**0.360**	
	9. Protein Comp	LDH	**−0.769**	
		Total protein	**0.670**	

### Multi-state model formulation

**Fig. 2:**
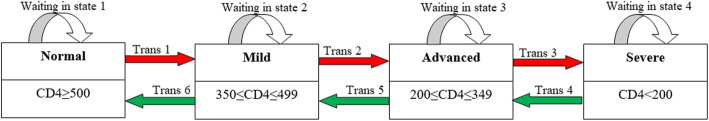
Progressive four-state model based on CD4 counts. Immunological recovery (green arrows), Immunological deterioration (red arrows) and waiting time (black and white arrows)

HIV infected, ART-naive patients may experience many CD4 cell count fluctuations, mostly before ART initiations. This indicates that the HIV disease process and progression should be modelled by a multi-state process. Figure 2 shows a flow diagram of the multi-state model. In this study, we considered six transitions: (1) Normal → Mild, (2) Mild → Advance, (3) Advance → Severe, (4) Severe → Advance, (5) Advance → Mild and (6) Mild → Normal. These mentioned transitions were modeled by a four-state multi-state transition-specific model. Attention was on predicting the probability of transition and the probability of staying in the same disease stage as well as examining the effects of possible factors on the transition intensities. We employed a multi-state model based on multicovariate parametric transition specific distribution.

A multi-state process is stated as using a Markov chain of {*X*(*t*), *t* ∈ *T*} that has finite space, denoted by *S* = {1, 2, 3, 4. ., *M*}. Here *T* = [0, *τ*] for *τ* < ∞. This Markov chains process has an initial probability, denoted by P(*X*(0) = *m*), *m* ∈ *S*, evolves over time and with a history (*H*_*s*_) which is containing the stage previously visited, and times of transitions [11, 28]. The multi-state process is differentiated through transition probabilities between two states *m* and *j* relative to the given process history is defined as

$$ {P}_{mj}\left(s,t\right)=P\left(X(t)=j|X(s)=m,{H}_s\right)\  for\ m,j\in S\  and\ s,t\in T,s<t\ (1) $$

where *P*_*mj*_(*s*, *t*) denotes the transition probability of the individual being in state *j* at time *t*, given that the individual was in state *m* at time *s* and ${\sum}_{j\epsilon S}{P}_{mj}=1$. If a transition probability not only depends on the current state *X*(*t*), but also on the entry time of the current state then the process is said to be a semi-Markov process [18]. Thus, the corresponding transition intensities (instantaneous hazard rate) is defined as follows

$$ {q}_{mj}(t)=\underset{\delta t\to 0}{\lim}\frac{P\left(X\left(t+\delta t\right)=j|X(t)=m\right)}{\delta t}\ (2) $$

Consequently, in our application, the *4 × 4* transition intensity matrix *Q*(*t*) is defined as

$$ \mathrm{Q}\left(\mathrm{t}\right)=\left[\begin{array}{cccc}-{q}_{12}(t)& {q}_{12}(t)& 0& 0\\ {}{q}_{21}(t)& -\left({q}_{21}(t)+{q}_{23}(t)\right)& {q}_{23}(t)& 0\\ {}0& {q}_{32}(t)& -\left({q}_{32}(t)+{q}_{34}(t)\right)& {q}_{34}(t)\\ {}0& 0& {q}_{43}(t)& -{q}_{43}(t)\end{array}\right] $$

Note that the rows sum to zero since ${\sum}_{j\epsilon S}{P}_{mj}=1$. The off-diagonal entries are the immunological deterioration and immunological recovery transition intensities). The diagonal entries are defined by ${q}_{mm}(t)=-{\sum}_{m\ne j}{q}_{mj}(t).$ The average length of stay in a single state before making any transitions to either a state associated with higher or lower CD4 count, is estimated by the negative inverse of the *m*^*th*^ diagonal entry of *Q*(*t*), that is $\frac{-1}{q_{mm}}$ . The amount of time spent (*L*_*j*_*)*, in-state *j* during the time period from *s* to *τ,* conditional on the patient being in state *m* at time *s* is defined as

$$ {L}_j={\int}_s^{\tau }{P}_{mj}(t) dt $$

In a multi-state process, the transition intensities of a patient moving to state *j* conditioned on state *m*, describes and characterizes the multi-state model [28]. A multi-state framework is stated as a combination of parametric transition-specific distributions [18]. The suitable way to do this is, using a “stacked data” representation [12]. Therefore, to examine the effect of covariates on such transitions, we defined a hazard function of the particular transition *m* → *j.*

$$ {q}_{mj}^i(t)={q}_0(t)\exp \left(\sum \limits_{k=1}^n{X}_k^i{\beta}_{kmj}\right)\ (3) $$

Where ${q}_{mj}^i$ represent the transition intensity of patient *i* from state *m* to state *j*, after adjusting a set of covariates. *q*_0_(*t*) is the baseline intensity and can be modelled parametrically and *β*_*kmj*_ is the log-linear effect of the kth covariate (${X}_k^i\Big)$ on the transition intensity ${q}_{mj}^i$. For this model transition where *m* > *j* is defined as immune recovery, then if *m* < *j*, it is defined as immune deterioration. Any standard survival model fitting software can be used to predict the transition hazard rate, once the cohort data is properly arranged. However, this implies that all transitions used in the same parametric survival model applies. In the next section, relaxing the above-mentioned assumption, allows each transition to use different potential parametric survival models, generating considerable flexibility in model building.

### Multi-state transition-specific parametric model

Since it is common to use the semi-parametric multistate model in modelling transitions intensity, there is a risk that if the PH assumption of some specific transition is not fulfilled, the results derived from this model will have a bias and will be flawed [29, 30]. Although some previous studies tend to turn a blind eye to this defect in their findings due to the ease of a semi-parametric multistate model application and its interpretation, it is essential to use alternative transition-specific parametric models with a higher degree of reliability for more precise investigations in such cases. Thus, in this paper, we present and compare different multi-state parametric transition-specific models.

Models can be fitted separately to each of the transitions. These can be more efficient computationally and allow the use of appropriate parametric models for each specific transition. This is also important as usually there would be sparse data for some specific transitions, and hence we could fit a simple parametric model (such as an exponential model) for a limited data transition, and a flexible parametric model (such as generalized Gamma, etc) for more information data transitions. This would allow an efficient approach to still make use of all medical cohort datasets [18]. Therefore, when allowing for arbitrary baseline hazard rates between each state, the above model (Eq. 3) generalizes to:

$$ {q}_{mj}^i(t)={q}_{mj,0}(t)\exp \left(\sum \limits_{k=1}^n{Z}_k^i{\beta}_{kmj}\right)\ (4) $$

Where *q*_0_(*t*) → *q*_*mj*, 0_(*t*), represents the baseline hazard function for a particular transition *m* → *j*, which allows taking appropriate parametric distribution approaches. In Eq. 3, we assumed a similar parametric model for all transitions. To keep flexibility, we have a vector of patient-level variables included in the particular transition *m* → *j,*${Z}_{mj}^i$, where ${Z}_{mj}^i\in Z$ (a set of available covariates). This also allows different variables to be included in different transitions. We present different distributional models including Exponential distribution, Weibull distribution, Log-logistic distribution, Log-normal distribution, and Generalized Gamma distribution. This allows for a variety of accelerated failure time models and proportional hazards models, emphasizing the flexibility of this structure.

The presence of time-varying effects is common in many health studies. For instance, in our HIV cohort data, where follow-up is usually over a long period, it is very important to examine the occurrence of time-varying effects. Including time-varying factors within a multi-state structure has received very little attention. In this current multi-state transition-specific parametric modelling method, we extend the structure to an accelerated failure time model which is used for allowing time-varying factors, in either separate modelling approaches or combined modelling approaches fitted to a “stacked data”. Therefore, one of the main advantage of the transition-specific parametric approach is an ease of incorporating time-dependent effects. Thus, the extended transition specific hazard function of the transition *m* → *j* is given by

$$ {q}_{mj}^i(t)={q}_{mj,0}(t)\exp \left(\sum \limits_{k=1}^n{Z}_k^i{\beta}_{kmj}(t)\right)\ (5) $$

Where *β*_*kmj*_(*t*) now allows to vary over time through some standard parametric distribution, relaxing the assumption of proportionality. For example, the baseline hazard function for a Weibull distribution is given by: *q*_*mj*, 0_(*t*) = *λγt*^*γ* − 1^. We can allow the independent variables in the linear predictor of the shape parameter (*γ*) on the log scale. Similarly, for the generalized gamma AFT model we can allow the three parameters to vary depending on the independent variables. So, with this flexibility, we can apply a parametric survival distribution with any specific transition containing time-varying variables and then, using one of the estimation techniques, we can estimate the parameters.

In this paper, in addition to comparing different parametric multi-state models in modelling transition intensity among different states, the estimates of the selected parametric multistate models were compared with the non-parametric estimate to assess model fit (as discussed by Ieva et al. [31] and Titman and Sharples [32]). The parameters are estimated by maximum likelihood.

### Prediction from non-parametric and parametric multistate models

To predict the probability of transitions and probability of staying in each disease state at a fixed time in the future, we calculated the probability of transition matrix *P*_*mj*_(*s*, *t*), where *P*_*mj*_(*s*, *t*) denotes the transition probability of the individual being in state *j* at time *t*, given that the individual was in state *m* at time *s (s < t)*. Under all models, this is calculated by simulating a large number of patients disease states histories from non-parametric and the transition-specific multistate models given the cumulative hazards or covariate-specific hazards for each transition. The implementation was carried out using Stata package (streg and multistate command) and R package (mstate and flexsurvreg codes).

## Results

### Baseline characteristics of the study population

Table 2 showed the characteristics of the demographic and clinical variables at the baseline of patients followed-up and the observed transitions. All participants were black women (*n* = 219), with a mean age of 26.67 years (standard deviation of 6.9 years). The majority of participants were overweight or obese 137 (62.8%), not with anaemia 208 (95.0%), not co-infected with TB 201 (91.8%), and married or with a stable partner 174 (79.5%). Over half 153 (69.9%) reported having completed Grades 11 of schooling. The maximum transition count was at a mild disease stage, recorded from normal disease stage at 447 (26.1%). The viral load of the participants ranged from 1.47 log_10_ copies/ml to 6.81 log_10_ copies/ml with the first quartile of 3.56 log_10_ copies/ml, a median of 4.23 log_10_ copies/ml and the third quartile of 4.79 log_10_ copies/ml.

**Table 2:** Baseline Socio-demographic and clinical characteristics in the CAPRISA 002 trials

Variables	Count/Mean (Percentage/SD)
**Marital Status, n(%)**	
Single/no partner	34(15.5)
Married/stable partner	174 (79.5)
Many partners	11 (5.0)
**Educational Status, n(%)**	
≤ Grade 8	16 (7.31)
Grade 9–10	50 (22.8)
≥ Grade 11	153 (69.9)
**Age Categories, n(%)**	
18–20 years	29 (13.2)
21–39 years	178 (81.3)
40–59 years	12 (5.5)
**BMI Categories, n(%)**	
Underweight	5 (2.3)
Healthy weight	76 (34.9)
Overweight/Obese	137 (62.8)
**Baseline immunologic state, n(%)**	
< 200 CD4 cells/mm3	9 (4.1)
200–349 CD4 cells/mm3	41 (18.7)
350–499 CD4 cells/mm3	89 (40.6)
≥ 500 CD4 cells/mm3	80 (36.5)
**Contraceptive use, n(%)**	
No	40 (18.3)
Yes	179 (81.7)
**Anaemia, n(%)**	
No	208 (95.0)
Yes	11 (5.0)
**TB Co-infection, n(%)**	
No	201 (91.8)
Yes	18 (9.2)
**Sex acts under the influence of alcohol, n(%)**	
No	197 (90.0)
Yes	22 (10.0)
**Viral load log**_**10**_**copies/ml, Mean (SD)**	4.46 (0.85)
**Number of transitions between stage, n(%)**	
Transition 1: Normal to Mild	447 (26.1)
Transition 2: Mild to Advance	374 (21.9)
Transition 3: Advanced to Severe	103 (6.03)
Transition 4: Severe to Advance	83 (4.9)
Transition 5: Advance to Mild	318 (18.9)
Transition 6: Mild to Normal	386 (22.6)

### Estimated probability of transitions and state-specific duration

**Fig. 3:**
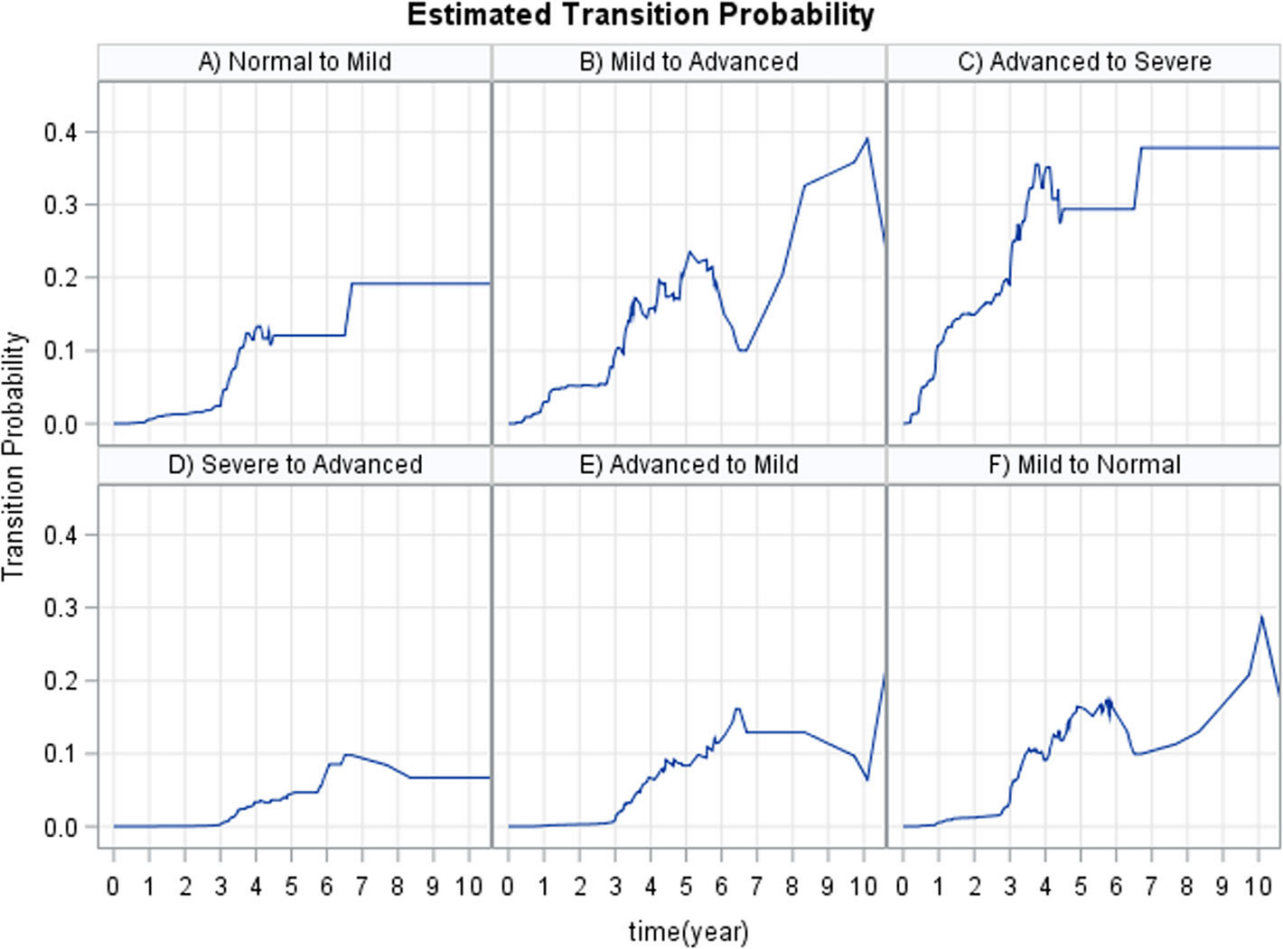
Estimated transition probability using Aalen-Johansen estimator. **a** The probability of transition from normal to mild disease state, **b** The probability of transition from mild to advance, **c** The probability of transition from advance to severe, **d** The probability of transition from severe to advance, **e** The probability of transition from advance to mild and F) The probability of transition from mild to normal disease state

**Fig. 4:**
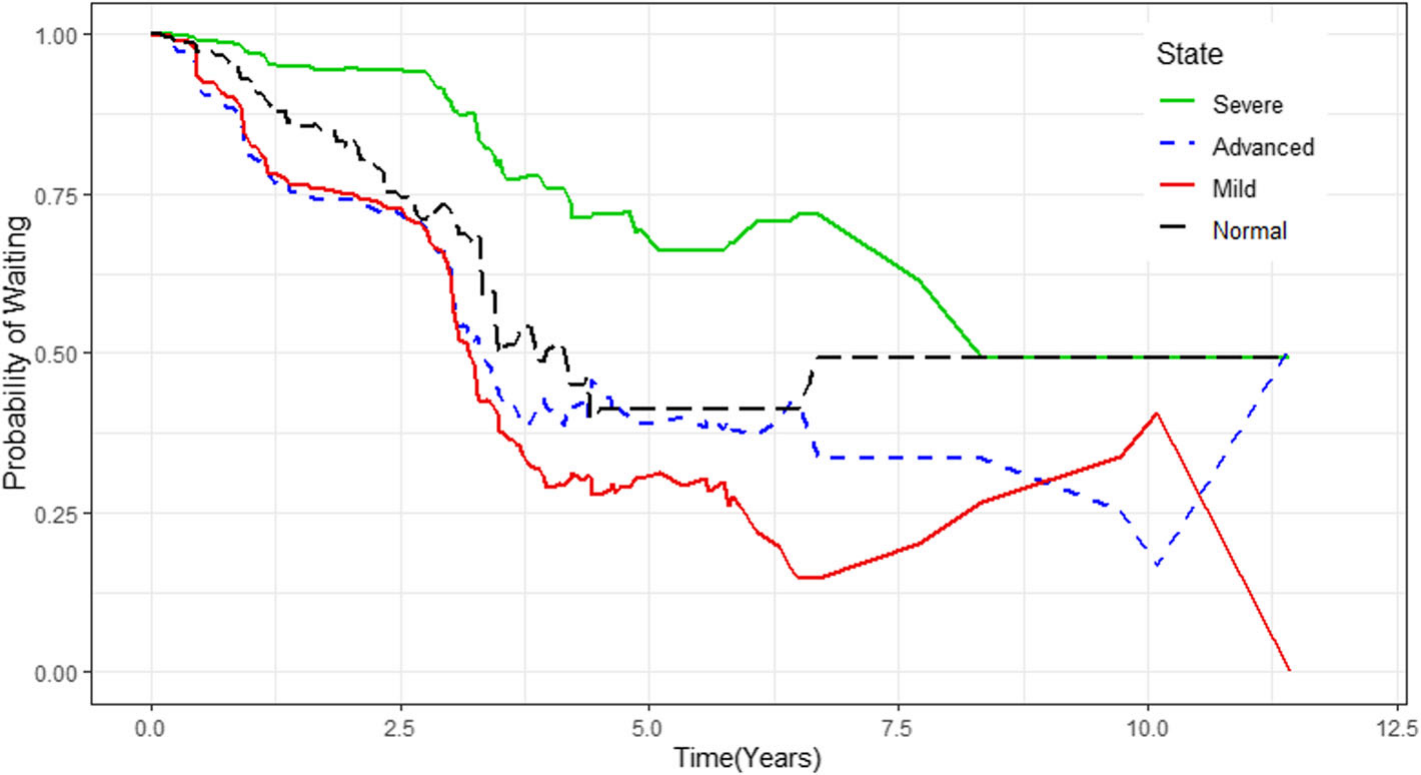
Estimated probability of staying in normal disease stage (black), mild disease stage (red), advance disease stage (blue) and severe disease stage (green)

The plot in Fig. 3(D-F) displays the probability of transitions from a state of lower CD4 cell count to a state of higher CD4 cell count (immune recovery) in HIV infected women. From this plot, we note that the probability of immune recovery (i.e. from advanced to mild, and severe to advanced stages) did not increase much, whilst the transition probability from a state of higher CD4 cell count, to a state of lower CD4 cell count (immune deterioration), increased with increasing years since entrance into a particular state (Fig. 3a-c). In other words, women who had enrolled with a CD4 cell count of less than 350 cells/mm^3^ (severe and advanced disease stage) had a far smaller chance on immune recovery, and a considerably greater chance of immune deterioration compared to women with a CD4 cell count of 350 cells/mm^3^ and more (mild and normal disease stage). The probability of staying in the same disease stage was also computed and represented graphically (see Fig. 4). From these plots, we further note that the probability of staying in the same disease state, decreased with increasing years following entrance into a particular state. The plot also showed that the probability of remaining in severe disease state over time was higher than remaining in other disease states.

### Results of transition-specific parametric multi-state model

We applied multi-state transition specific parametric distribution, such as the generalized Gamma, Exponential, Log-logistic, Weibull and Log-Normal models. In Table 3, we present the model selection criteria for each transition and model fitted. We found that the best parametric fitting distribution for transition 4 was the log-normal distribution, based on model selection criteria. The best parametric fitting distribution for transition 1, 2, 3 and 5 were found to be the Log-logistic model, based on model selection criteria. Similarly, the best parametric fitting distribution for transition 6 was found to be the Weibull model, based on the BIC and AIC. Furthermore, we included all possible demographic and clinical variables and assessed the assumptions. For instance, we fitted a Cox regression to each specific transition and then used the interaction between logarithm transformation of time and each explanatory variable to test for proportionality. For each transition, there is a violation of the proportional hazards assumption. Thus, to incorporate time-dependent effects, we used accelerated failure time models.

**Table 3:** Model selection criteria for each parametric model to each transition separately

Transition	Criteria	Exp.	Weibull	Log-logistic	Log-Normal	Gen-Gamma
1	AIC	2021.85	1880.14	**1867.61**	2114.48	1868.15
	BIC	2168.97	2032.71	**2020.17**	2270.56	2026.17
2	AIC	1992.44	1841.26	**1822.15**	1887.68	1824.26
	BIC	2143.07	1997.47	**1978.35**	2040.24	1986.04
3	AIC	690.28	638.59	**633.21**	636.67	637.12
	BIC	825.39	778.69	**773.32**	776.78	782.23
4	AIC	341.37	322.52	375.61	**321.32**	323.16
	BIC	426.98	411.29	470.90	**410.09**	415.09
5	AIC	1606.12	1538.31	**1524.84**	1528.19	1527.79
	BIC	1741.73	1678.95	**1665.48**	1668.82	1673.46
6	AIC	2185.01	**2109.77**	2117.94	2114.48	2117.64
	BIC	2335.51	**2265.84**	2265.01	2270.55	2289.44

Results of the multi-state model (see Table 4) showed that hemoglobin abnormality had a significant effect on the time lag for improving from severe to advanced stages of the disease. The interpretation is that the recovery from severe to advanced disease was delayed to a relative rate of 0.71 in those who were anaemic compared with those with normal hemoglobin (aTR = 0.71, 95% CI:0.55–0.92). Having a high VL, significantly accelerated the deterioration from normal to mild (aTR = 1.26, 95% CI:1.19–1.33), mild to advanced (aTR = 1.27, 95% CI:1.20–1.35) and advanced to severe disease states (aTR = 1.32, 95% CI:1.18–1.48). Similarly, as the viral load of women increased, the recovery from advanced to mild (aTR = 0.95, 95% CI:0.89–0.98) and mild to normal disease stage (aTR = 0.94, 95% CI:0.89–0.99) was delayed to a relative rate of 0.95 and 0.94, respectively. Patients in the middle-aged group were significantly decelerating the recovery from severe to advanced (aTR = 0.90, 95% CI:0.83–0.98) disease stage compared with those patients in the older-aged group. Patients who reported many sex partners were significantly decelerating the recovery from severe to advanced stages (aTR = 0.71, 95% CI:0.65–0.78) compared with those patients with no sexual partner (single). Moreover, patients with high liver enzyme abnormalities significantly decelerated the recovery from advance to mild stage of the disease (aTR = 0.93, 95% CI:0.87–0.98) for the HIV infected patients in the study.

**Table 4:** Estimates and the 95% confidence intervals for parameters of multistate transition-specific parametric models

Variables	Transition 1: Normal to Mild, TR(95% CI)	Transition 2: Mild to Advanced, TR (95% CI)	Transition 3: Advanced to Severe, TR (95% CI)	Transition 4: Severe to Advanced, TR (95% CI)	Transition 5: Advanced to Mild, TR (95% CI)	Transition 6: Mild to Normal, TR (95% CI)
Viral Load	**1.26 (1.19, 1.33)**^a^	**1.27 (1.20, 1.35)**^a^	**1.32 (1.18, 1.48)**^a^	1.16 (0.98, 1.37)	**0.95 (0.89, 0.98)**^a^	**0.94 (0.89, 0.99)**^b^
Anaemia: Yes	1.01 (0.91, 1.13)	1.09 (0.99, 1.20)	1.23 (1.03, 1.48)	**0.71 (0.55, 0.92)**^a^	0.94 (0.82, 1.06)	0.92 (0.82, 1.03)
TB: No	1.11 (0.83, 1.50)	1.19 (0.98, 1.44)	1.07 (0.69, 1.68)	**1.15 (1.09, 1.22)**^a^	0.92 (0.73, 1.16)	**1.50 (1.13, 2.01)**^a^
Marital Status: Married	1.01 (0.88, 1.16)	**0.80 (0.70, 0.90)**^a^	0.96 (0.75, 1.23)	0.91 (0.66, 1.27)	0.92 (0.80, 1.07)	0.96 (0.84, 1.10)
Marital Status: Many Partners	1.12 (0.89, 1.42)	0.74 (0.59, 1.09)	0.78 (0.53, 1.14)	**0.71 (0.65, 0.78)**^a^	0.92 (0.72, 1.17)	0.98 (0.75, 1.26)
Education: Grade 9–10	**0.74 (0.61, 0.89)**^a^	0.95 (0.80, 1.12)	1.15 (0.87, 1.51)	1.09 (0.69, 1.73)	0.97 (0.79, 1.18)	1.06 (0.87, 1.29)
Education: Grade ≥ 11	**0.82 (0.69, 0.99)**^a^	0.84 (0.72, 1.04)	0.81 (0.63, 1.04)	1.02 (0.68, 1.51)	0.96 (0.81, 1.15)	1.10 (0.93, 1.32)
Contraceptive use: No	1.03 (0.91, 1.16)	0.99 (0.89, 1.11)	1.02 (0.83, 1.25)	0.99 (0.73, 1.34)	**0.85 (0.74, 0.97)**^a^	0.89 (0.78, 1.01)
Age_Cat: ≤20 years	1.08 (0.95, 1.22)	1.04 (0.92, 1.18)	0.99 (0.78, 1.24)	1.12 (0.78, 1.60)	1.15 (0.99, 1.34)	1.03 (0.91, 1.17)
Age_Cat: 21–39 years	1.11 (0.89, 1.38)	0.87 (0.68, 1.11)	0.74 (0.44, 1.24)	**0.90 (0.83, 0.98)**^b^	1.42 (0.96, 1.94)	1.33 (0.94, 1.65)
Sex while Drunk: No	1.04 (0.89, 1.21)	0.88 (0.75, 1.04)	**0.76 (0.60, 0.98)**^a^	**1.07 (1.01, 1.12)**^a^	0.88 (0.74, 1.04)	1.01 (0.85, 1.21)
Weight	**0.99 (0.98, 1.01)**^a^	**0.99 (0.97, 1.02)**^a^	1.01 (0.99, 1.01)	**1.01 (1.00, 1.02)**^a^	1.00 (0.99, 1.01)	1.00 (0.99, 1.01)
Independence Score	**0.95 (0.94, 0.96)**^a^	0.99 (0.97, 1.01)	**0.97 (0.94, 0.99)**^a^	0.99 (0.94, 1.03)	1.00 (0.99, 1.02)	1.01 (0.99, 1.02)
Social relationship score	**0.98 (0.96, 0.99)**^a^	0.99 (0.97, 1.01)	**0.96 (0.93, 0.99)**^a^	0.99 (0.94, 1.07)	1.00 (0.98, 1.02)	0.96 (0.95, 1.02)
Physical Health Score	1.13 (0.92, 1.16)	1.03 (1.00, 1.06)	1.01 (0.97, 1.07)	**1.09 (1.02, 1.16)**^a^	1.01 (0.97, 1.03)	**1.07 (1.04, 1.10)**^a^
Psychological Score	**0.90 (0.88, 0.93)**^a^	**0.95 (0.93, 0.97)**^a^	**0.95 (0.90, 0.99)**^a^	0.94 (0.88, 1.01)	0.95 (0.92, 1.03)	0.94 (0.92, 1.04)
Liver Abn Comp	0.97 (0.94, 1.02)	0.99 (0.95, 1.04)	1.01 (0.94, 1.08)	1.00 (0.89, 1.12)	**0.93 (0.87, 0.98)**^a^	1.03 (0.99, 1.08)
RBC indices	0.98 (0.94, 1.03)	**0.95 (0.91, 0.99)**^b^	0.99 (0.91, 1.08)	0.96 (0.85, 1.07)	0.94 (0.89, 1.00)	1.04 (1.00, 1.10)
Hb and Haematocrit Comp	0.97 (0.92, 1.01)	0.99 (0.95, 1.04)	1.01 (0.94, 1.10)	1.04 (0.93, 1.16)	1.01 (0.96, 1.07)	1.04 (0.99, 1.09)
Granulocytes comp	0.95 (0.92, 1.00)	0.95 (0.90, 1.01)	0.99 (0.91, 1.08)	1.01 (0.88, 1.15)	**1.08 (1.03, 1.14)**^a^	1.05 (1.00, 1.10)
Mononuclear comp	0.87 (0.83, 1.09)	**0.82 (0.78, 0.87)**^a^	**0.82 (0.74, 0.91)**^a^	1.12 (0.98, 1.27)	**1.14 (1.08, 1.18)**^a^	**1.18 (1.12, 1.23)**^a^
Eosinophils comp	1.01 (0.97, 1.05)	0.97 (0.93, 1.01)	1.01 (0.92, 1.11)	0.97 (0.89, 1.05)	0.98 (0.93, 1.03)	1.00 (0.96, 1.04)
Electrolyte Comp	0.98 (0.94, 1.03)	1.01 (0.97, 1.06)	1.05 (0.97, 1.14)	0.94 (0.85, 1.05)	1.04 (0.99, 1.10)	1.04 (0.99, 1.08)
Protein Comp	0.96 (0.91, 1.01)	1.03 (0.98, 1.08)	0.99 (0.90, 1.09)	0.93 (0.83, 1.05)	1.03 (0.97, 1.09)	1.00 (0.96, 1.04)
Intercept	**0.40 (0.22, 0.71)**^a^	**0.43 (0.27, 0.70)**^a^	**0.23 (0.08, 0.60)**^a^	**0.21 (0.05, 0.92)**^a^	1.01 (0.57, 1.76)	**0.35 (0.20, 0.61)**^a^

**Keys**:- (^a^) the coefficient is significant at α = 0.05, (^b^) the coefficient is a significant at α = 0.01, Reference category: Age [> 40 years], education [≤ Grade 8], marital status [Single], Sex while Drunk [Yes], Contraceptive use [Yes], Anemia [No], and TB [Yes]

With regard to factors that accelerate recovering or decelerate deterioration, patients without TB co-infection were significantly associated with a shortened time to immunological recovery, compared to those with TB co-infection (particularly from severe to advanced (aTR = 1.51, 95% CI:1.09–1.22) and normal to mild disease stages (aTR = 1.50, 95% CI:1.13–2.01)). Having a high physical health score, significantly accelerated the recovery from severe to advance (aTR = 1.09, 95% CI:1.02–1.16) and from mild to normal disease stage (aTR = 1.07, 95% CI:1.04–1.10). Having a high level of independence score, significantly decelerated the deterioration from normal to mild (aTR = 0.95, 95% CI:0.94–0.96), and also from advanced to severe disease stage (aTR = 0.97, 95% CI:0.94–0.99). Similarly, having a high social relationship score significantly decelerated the deterioration from normal to mild and from advanced to severe disease stages. As the psychological wellbeing score increased, the deterioration from advanced to severe, from mild to advanced, and from normal to mild stages, was decelerated to a relative rate of 0.95, 0.95, and 0.90, respectively.

Patients with a stable sexual partner, were found to be associated with decelerating immune deterioration from mild to advanced disease stages compared with those with no sex partner. Patients with higher educational levels, were associated with a longer time to immunological deterioration (particularly from normal to mild disease stages), compared with those women with lower educational levels. Moreover, having a high weight significantly decelerated the deterioration from mild to advanced disease stage (aTR = 0.98, 95% CI:0.97–0.99) and from normal to mild disease stage (aTR = 0.98, 95% CI:0.97–0.99). The time for transition from advance to severe disease stages (aTR = 0.76, 95% CI:0.60–0.98), of women in the study who did not have sex under the influence of alcohol, was decelerated by a factor of 0.76, as compared to those who had sex under the influence of alcohol.

Having a high RBC indices score significantly decelerated the deterioration from mild to advance disease stage (aTR = 0.95, 95% CI:0.91–0.99). Similarly, we noted that having a high mononuclear score significantly accelerated the recovery time from advanced to mild and from mild to normal disease stages, but significantly decelerated the deterioration from normal to mild and from advanced to severe stages of the disease. Finally, we noted that as the latent variable related to granulocytes increased, the time for transition from advanced to mild stages of the disease was accelerated by a factor of 1.08 (aTR = 1.08, 95% CI:1.03–1.14).

### Assessment of goodness of fit of the model

**Fig. 5:**
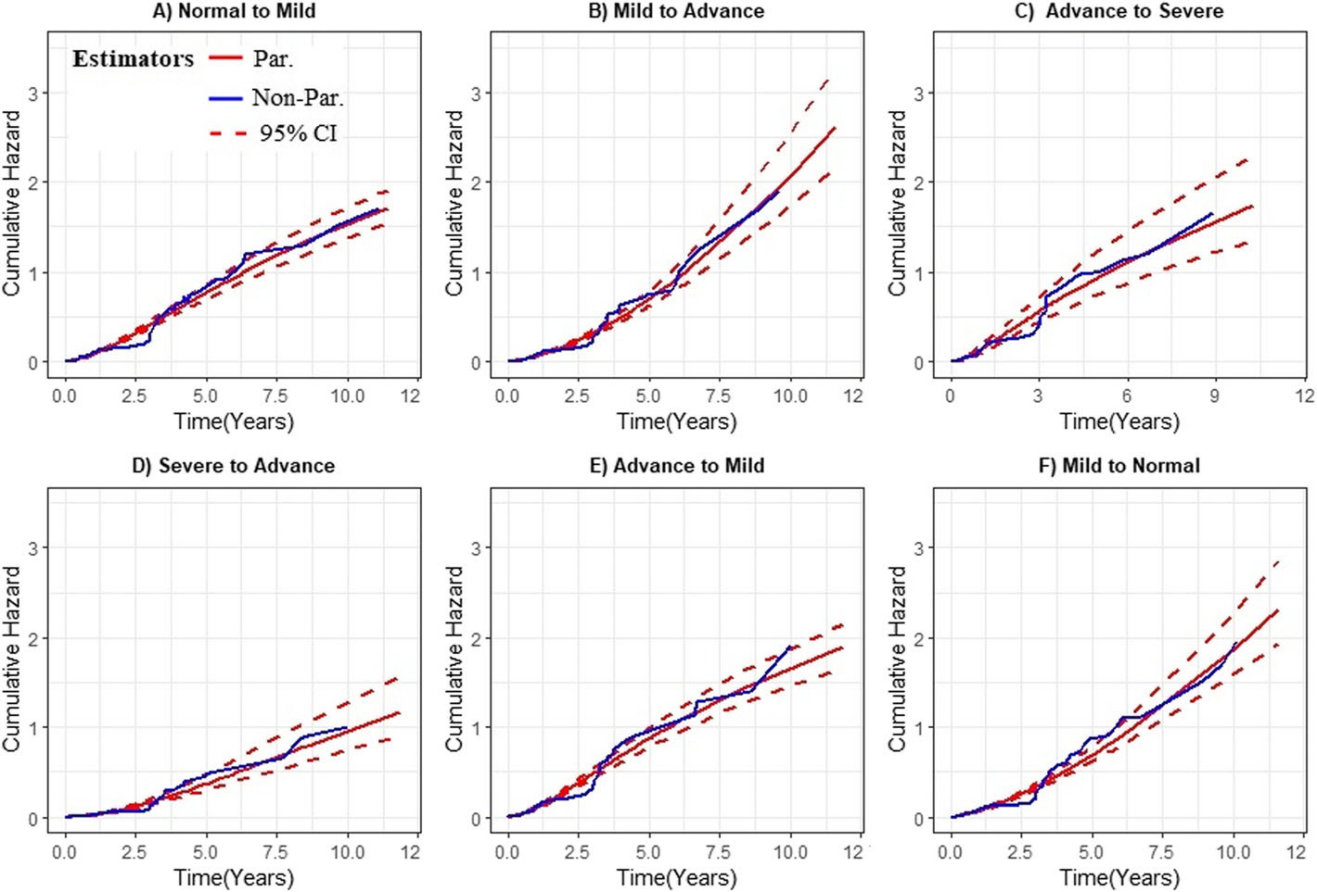
Goodness-of-fit plots. **a**, **b**, **c** and **e**) Log-logistic cumulative hazard curve (red solid line) and its 95% CI (red dotted line) overlaid on non-parametric estimates (blue solid line), **d** Log-normal cumulative hazard curve (red solid line) and its 95% CI (red dotted line) overlaid on non-parametric estimates (blue solid line) and **f**) Weibull cumulative hazard curve (red solid line) and its 95% CI (red dotted line) overlaid on non-parametric estimates (blue solid line)

The estimates of the transition specific parametric multistate model were validated by using graphical methods presented in Fig. 5. The estimates of these multistate models were compared with a non-parametric estimate to assess model fit. The fitted cumulative hazard functions for each of the transitions (obtained from the transition specific parametric multistate model), are shown in Fig. 5(A-F), overlaid on the nonparametric estimator of the transitions (Aalen-Johansen estimator). These plots showed the overall good performances of our transition-specific multi-state models (ie. log-normal model for transition 4, log-logistic model for transition 1, 2, 3 and 5 and Weibull model for transition 6), in terms of fit for the transitions cumulative hazard estimate.

## Discussion and conclusion

Intermediate endpoints play a significant role in HIV disease process and progression in many survival analysis studies. Using separate statistical analyses for every single event is the usual approach in many medical studies [33], but does not give the possibility of detecting associations between the endpoints [34, 35]. Utilizing the multi-state statistical model improves understanding of the variation in the possible factors related to HIV disease process and progression [11]. With the increasing use of electronic medical cohort data, allowing the integration of clinical registers and organizational records, there will be considerable opportunities to use multi-state models for expanded modelling of patient profiles across disease progression histories [36]. This has an advantage not only for clinical efficiency modelling, but also for the efficiency of cost modelling due to the parametric methods [37].

Within a multi-state structure, parametric modelling approaches have usually considered distributions that assume the same model for all transitions. In this current work, we have presented a relatively new and under-utilized method for analyzing time to sequential ordinal response variables. We presented the use of the multi-state parametric transition-specific model, allowing a combination of parametric models (such as proportional hazards and accelerated failure time). We then used an Aalen-Johansen estimator to estimate the probabilities of transitions and probability of staying in the same disease stage. Furthermore, we introduced a structure to allow the flexibility of multi-state transition-specific parametric AFT models, but still enabling the sharing of parameters across the transitions.

The results also showed that patients with higher educational levels were significantly associated with decelerating immune deterioration compared to those patients with lower educational levels. Our finding is concurrent with those from prior reports [38, 39], which noted that patients having a higher educational level significantly associated with a better rate of change of immunological recovery. This might be due to literate patients having work capacity, financial resources, and access to quality health care. Patients in the middle-aged group were significantly decelerating time to immunological deterioration compared with those older patients, a finding that is in accordance with the literature [40, 41], which noted that middle-aged adults experienced higher rates of CD4 recovery than the elderly. Furthermore, patients with many sex partners were significantly decelerating the recovery from severe to advanced stages compared with those with no sexual partner. As has been previously shown [42], patients with higher incidences of sexual risk-taking behavior (such as many sex partners) were significantly associated with low QoL and chronic depression of HIV patients. Chronic depression and low QoL scores are significantly linked to lower CD4 cell count [43, 44], showing that the effect of many sex partners on incomplete immune recovery, is mediated through depression and QoL.

We have also found that patients with high *scores* in *quality* of *life* significantly accelerated the immune recovery time, but significantly decelerated the immune deterioration time of HIV infected patients. This was supported by studies in South Africa: Venter et al. [45] and Ingumbor et al. [46] have found a significant positive association between trends of CD4 cell count recovery and health-related QoL scores of HIV infected patients.

Among the different clinical attributes of patients, patients having higher mononuclear scores significantly accelerated the recovery from advanced to mild and from mild to normal disease stages but significantly decelerated the deterioration from normal to mild and from advanced to severe stages of the disease. Our finding is concurrent with those from study [47], which observed that the increase in the *basophils counts and total lymphocytes counts* corresponded to an increase in the CD4 cell count. We also observed that patients with high liver enzyme abnormalities significantly decelerated the recovery from advance to mild stage of the disease. A previous study also reported a similar finding [48], which observed that lower CD4 count was found to be associated with elevated ALT and AST. Thus, there is a need to monitor ALT and AST levels of the patients before the initiation of cART to reduce side effect concerns. Moreover, having high scores of red blood cell latent and higher granulocytes scores had a significant effect on a shortened time to immunological recovery.

Finally, it can be concluded that transition-specific distributions for multi-state modelling offer a flexible tool for the study of covariate effects on the various transition rates. These models may reveal important biological insights that could otherwise be overlooked when using a model for the marginal survival distribution. The tools are available in terms of methods and software, so hopefully, this paper has helped researchers familiarize themselves with some of these model approaches as well as with the interpretation of multi-state model results (particularly for medical research). In future work, we plan to develop a joint model for multivariate longitudinal biomarkers and multi-state processes in HIV/AIDS diseases on some relevant factors, covariates and a set of latent variables.

## Data Availability

The dataset used and analyzed during the current study is available from the corresponding author on reasonable request.

## Abbreviations

*AIDS*: Acquired Immune Deficiency Syndrome
ALT: Alanine aminotransferase
ART: Antiretroviral therapy
*ARV*: Antiretroviral drug
AST: Aspartate aminotransferase
BP: Blood pressure
CAPRISA: Center of the AIDS program of research in South Africa
HB: Hemoglobin
HIV: Human Immunodeficiency Virus
LDH: Lactate dehydrogenase
MCH: Mean corpuscular hemoglobin
MCHC: Mean corpuscular hemoglobin concentration
MCV: Mean corpuscular volume
OI: *Opportunistic infections*
PLHIV: people living with HIV
PR: Pulse rate
QoL: Quality of life
RBC: Red blood cells
RDW: Red cell distribution width
V B12: Vitamin B12
